# Serum metabolomics approach to monitor the changes in metabolite profiles following renal transplantation

**DOI:** 10.1038/s41598-020-74245-z

**Published:** 2020-10-14

**Authors:** Ivana Stanimirova, Mirosław Banasik, Adam Ząbek, Tomasz Dawiskiba, Katarzyna Kościelska-Kasprzak, Wojciech Wojtowicz, Magdalena Krajewska, Dariusz Janczak, Piotr Młynarz

**Affiliations:** 1 Institute of Chemistry, The University of Silesia, 9 Szkolna Street, 40-006 Katowice, Poland; 2 Department of Nephrology and Transplantation Medicine, Wroclaw Medical University, 213 Borowska Street, 50-556 Wroclaw, Poland; 3 PORT Polish Center for Technology Development, 147 Stablowicka Street, 54-066 Wroclaw, Poland; 4 Department of Vascular, General and Transplantation Surgery, Wroclaw Medical University, 213 Borowska Street, 50-556 Wroclaw, Poland; 5 Department of Bioorganic Chemistry, Wrocław University of Technology, 27 Wybrzeze Wyspianskiego Street, 50-370 Wroclaw, Poland

**Keywords:** Chronic kidney disease, Metabolomics

## Abstract

Systemic metabolic changes after renal transplantation reflect the key processes that are related to graft accommodation. In order to describe and better understand these changes, the ^1^HNMR based metabolomics approach was used. The changes of 47 metabolites in the serum samples of 19 individuals were interpreted over time with respect to their levels prior to transplantation. Considering the specific repeated measures design of the experiments, data analysis was mainly focused on the multiple analyses of variance (ANOVA) methods such as ANOVA simultaneous component analysis and ANOVA-target projection. We also propose here the combined use of ANOVA and classification and regression trees (ANOVA-CART) under the assumption that a small set of metabolites the binary splits on which may better describe the graft accommodation processes over time. This assumption is very important for developing a medical protocol for evaluating a patient’s health state. The results showed that besides creatinine, which is routinely used to monitor renal activity, the changes in levels of hippurate, mannitol and alanine may be associated with the changes in renal function during the post-transplantation recovery period. Specifically, the level of hippurate (or histidine) is more sensitive to any short-term changes in renal activity than creatinine.

## Introduction

Chronic kidney disease (CKD) is a global public health issue^1–3^ with an estimated prevalence of 8–16% worldwide. The state of CKD is recognized as either a kidney function decrease with a glomerular filtration rate of less than 60 mL/min per 1.73 m^2^, or as a presence of the kidney damage markers such as structural abnormalities or albuminuria, or as a presence of both conditions for at least three months^4–8^. The frequency of the disease depends on its two most common causes, which are diabetes and hypertension in all high- and middle-income countries^5,6^. Some other possible causes of CKD that include glomerulonephritis, polycystic kidney disease, obstructive uropathy, vesicoureteral reflux, recurrent pyelonephritis, and the chronic use of some medications, are less common^6^. The devastating complications of CKD include an increased incidence of cardiovascular disease, hyperlipidemia, anemia and osteodystrophy^7^, which cause a decreased quality of life, but most importantly, which are associated with premature mortality^2^. According to the Global Burden of Disease Study ranking, CKD was the 12th most common cause of death globally in 2015^8,9^.

End-stage renal disease (ESRD) patients require a dialysis therapy or renal transplantation in order to survive^6^, however, transplantation is always the best therapeutic option for them^10–12^. Successful kidney transplantation doubles^11^ or even triples^12^ the life expectancy of the ESRD patients that are listed for transplantation, while, the annual death rate for all patients on dialysis is more than ten times higher compared to the transplant recipients^11^. Despite the significant improvement in the results of kidney transplantation, which are expressed by more than 90% of 1-year grafts survival, still only about 50% of patients preserve graft function for more than 10 years^13^. Many immunological and non-immunological factors are responsible for this and not all of them are known yet^14–17^. Creatinine is routinely used in clinical practice as an indicator of kidney dysfunction, but it has been reported^18^ that its level becomes perturbed only as a result of strong kidney damage when a therapeutic intervention may be ineffective. Up to 30% of grafts with stable creatinine may also experience ‘smoldering’ rejection^19^. Moreover, determining the levels of immunosuppressants in serum also seems not to be sufficient enough, and a renal biopsy is too invasive. It is not surprising then that transplantologists are still looking for some additional indicators, e.g. some possible biomarkers in order to improve the post-transplantation results. A technique that may help to increase the knowledge about the mechanisms of graft adaptation and its insufficiency and may possibly be useful for developing a non-invasive method for transplant monitoring is metabolomics. The reason is that changes in the concentrations of metabolites in the blood samples of graft recipients reflect the changes in graft function, the processes of humoral or cellular rejection, immunsouppressant toxicity or in other factors over time. NMR spectroscopy is a powerful analytical method for obtaining comprehensive profiles of the metabolite signals without separating, derivatizing and pre-selecting the measurement parameters. Because of its high degree of reproducibility and suitability in clinical settings, NMR spectroscopy has been preferred for monitoring the metabolite changes that occur in urine samples from patients with kidney transplants^20^ (it is also the technique of choice in our study). In general, in order to describe and interpret the biochemical changes that are associated with the progress of recovery over time, the multivariate data analysis that is required for this purpose should reflect the specific dynamic structure of the data^21^. Traditionally, principal component analysis (PCA) has been used to examine whether there are some patients that have different recovery profiles and to identify the metabolites that are responsible for these differences, while the discriminant variant of partial least squares regression (PLS-DA) has been used to interpret the before-after transplantation changes by polling the individual metabolic profiles of a large number of patients in one of these two groups. Both methods explain the total variation in the data, and this may not be optimal when the analysis is focused more on modeling the individual variation of patients or when a specific design of experiments is assumed. For example, to monitor transplant patients in a short (2-week) recovery period, a strategy combining the modeling of the NMR measurement data of the urine samples that were collected from each patient using the orthogonal projection to latent structures (OPLS) approach and comparing each individual OPLS effect profile, which is associated only with the individual metabolic variation over time, with the profiles of other patients using PCA, has been proposed. This strategy is specifically oriented towards determining whether a patient is moving towards recovery or whether some health complications can be expected^22^.

In this study, we attempt to identify which serum metabolites are most important when describing the short- or longer-term (up to 6 months) graft accommodation. For this purpose, the levels of 47 metabolites in the serum samples of 19 patients that had a renal transplant were monitored over time. All of the metabolite changes are typical for the normal recovery process. Taking into account the repeated measures structure of the data, the focus of our methodology for analysis is on explaining the metabolic changes across all patients over time and interpreting the inter-patient metabolic changes. Specifically, using the repeated measures analysis of variance (ANOVA) enabled us to partition the total variability of the data for each serum metabolite into a variability associated with the recovery progress over time, variability due to the individual differences among patients and error variability. Next, the collection of factor mean estimates for each serum metabolite can be analyzed using multivariate methods such as PCA, simultaneous component analysis (SCA), discriminant partial least squares regression (PLS-DA or with PLS-DA followed by the target projection method, TP^23^. Multiple ANOVA methods such as ANOVA-PCA^24^, ASCA^25^, AoV-PLS^26^ or ANOVA-TP^27^ have gained popularity in metabolomics in recent years. The latter ANOVA-PLS method enables the variables that are important to be selected using the selectivity ratio, SR, approach. The SR values for metabolites are evaluated after the target projection or target rotation transformation of the PLS components, which ensures that only metabolites that are related to the modeled groups are selected. The same objective is met by OPLS using a different algorithm. The main assumption in using multivariate methods for selecting metabolites is that a linear combination of metabolites is responsible for the differences in modeled groups. However, there might be a small set of metabolites, the orthogonal binary splits on which at given threshold values can form mutually exclusive regions in the data space, that contain as homogeneous groups of samples as possible. Binary data splitting is obtained from the classification and regression trees (CART^28^) method and its results are visualized as a binary tree, which contains a number of nodes associated with the data subgroups. The results are easily interpreted and can serve to create a diagnostic specification scheme for successfully determining a patient’s health state. Here, we also propose the combined use of ANOVA and CARTfor identifying the metabolites that are responsible for the short- or long-term before-after transplantation variations.

We are convinced that a comparison of the results from the above-mentioned methods will give useful information about the possible set of metabolites that can be used to predict the progress of recovery. It is also important to be able to asses whether the metabolites that are characteristic for a short recovery period can also be used for the longer-term monitoring of kidney function.

## Results

### Investigating metabolic changes across all of the individuals over time

In order to gain insight into the data structure, the NMR data were organized in a two-way table as (19 *individuals* × 4 *time points*) × 47 *metabolites* of the dimensions 76 × 47 and were PQN-normalized and centered. Serum samples were collected before renal transplantation (defined as ‘T0’) and after the transplant surgery at three time points defined as time ‘T1’ (1 day), ‘T2’ (7–10 days) or ‘T3’ (6 months) after transplantation. The time intervals (with an extension of the monitoring time) were set in agreement with our previous findings on how the post-transplantation metabolomic recovery processes can be controlled in patients.

**Figure 1:**
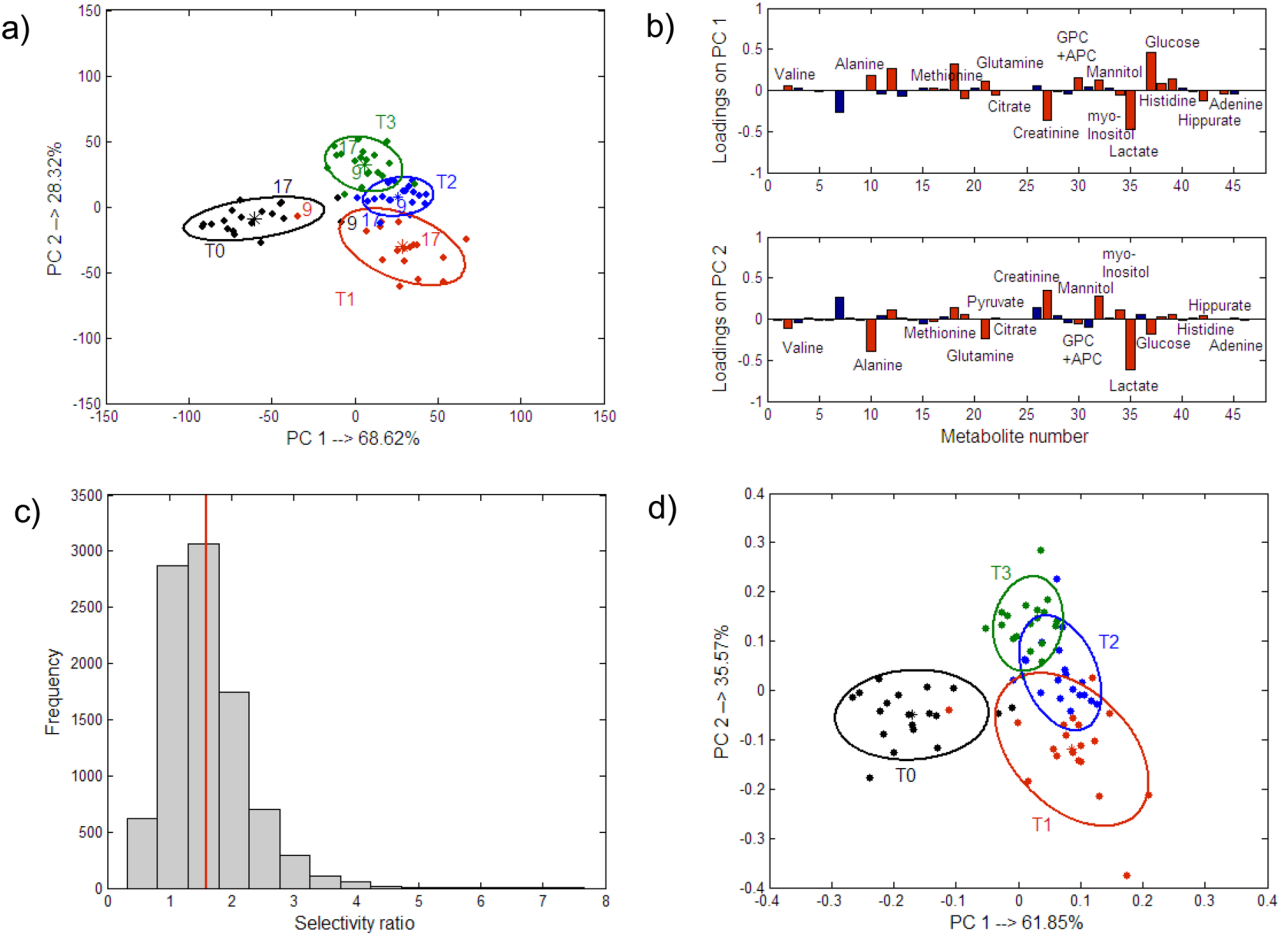
Projection of the samples in the space spanned by the first two latent components that were obtained from (**a**) ANOVA-TP using all of the metabolites along with the 90% confidence ellipses, which were placed at the centroids of the groups that were defined according to the ‘time’ factor, (**b**) The respective projections of metabolites on the first two PCs that were obtained from the ANOVA-TP. The most important metabolites are indicated by red bars. All of the metabolites with the mean selectivity ratio values, which were estimated by the bootstrapping procedure and were larger than 1.0 are finally considered being important. (**c**) Histogram constructed for the selectivity ratio values for the pyruvate metabolite that were obtained from 10,000 bootstrapped samples. The vertical red line illustrates the mean value of the selectivity ratio (mean SR = 1.6), which is also listed in Table 1. and (**d**) Projection of the samples in the space spanned by the first two latent components that were obtained from ANOVA-TP using selected metabolites.

As was mentioned earlier, with PCA, the total variance in the experimental data is explained and this might not be optimal for the highly structured data that are obtained from a time-course experiment. For a better description of the ‘time’ effect, the multiple ANOVA methods were used for the analysis. The main idea of these methods is to split the total variation of each metabolite (each column of the original PQN-transformed matrix) according to the repeated measures ANOVA design and to analyze the respective factor matrices using multivariate methods. A comparison of the results from PCA and the ANOVA-PCA or ASCA method for the ‘time’ effect is presented in Supplementary Sect. 2 of the supplementary material of this article. Compared to PCA, in ANOVA-PCA and ASCA, there was a clearer distinction between the groups of samples before and after transplantation and among the groups of samples from the entire post-transplantation period when the discriminant partial least squares regression, PLS-DA, combined with the target projection approach was used to model the factor matrix on the reduced residuals, e.g. ANOVA-TP. The residual matrix that is summed with the factor ‘time’ matrix does not contain the inter-individual variation, which helps in describing the within-group variation better. The best representation of the within-group variance that was obtained from ANOVA-TP is shown in Fig. 1a along with the respective 90% confidence ellipses for the centroids. The projections are color-coded according to the experimental time points. The respective target transformation loadings are presented in Fig. 1b. The most important metabolites are indicated in red and their mean concentration levels over time are listed in Table 1. They were selected as those that had mean selectivity ratio values larger than 1.0. The selectivity ratio value was calculated for each metabolite as the ratio between the metabolite variance that was explained by the PLS-DA model of a selected complexity and its residual variance after the target projection transformation. This means that the larger value of this ratio than 1.0, the greater importance of a metabolite was. A bootstrapped procedure with a block replacement was used to estimate the average selectivity ratio and its uncertainty. An exemplary histogram that was constructed for 10,000 selectivity ratio values from the block bootstrapping procedure for the pyruvate metabolite is shown in Fig. 1c. The vertical red line indicates the average selectivity ratio value, which in this case is equal to 1.6. The projections of the samples that were represented only with the selected variables are presented in Fig. 1d.

**Table 1:** Multivariate and descriptive statistics for the NMR variables that were found to be most important using the ANOVA-TP approach.

Metabolites	ANOVA-TP (all time points) Mean SR (≥ 1.0) ± SD	Mean contents of metabolites ± SD				ANOVA-TP T0/T1 (mean SR (≥ 1.0) ± SD)	ANOVA-TP T0/T2 (mean SR (≥ 1.0) ± SD)	ANOVA-TP T0/T3 (mean SR (≥ 1.0) ± SD)	ANOVA-TP T1/T3 (mean SR (≥ 1.0) ± SD)
		T0	T1	T2	T3				
Valine	1.5 ± 0.8	27.26 ± 6.44	31.31 ± 4.51	31.70 ± 5.39	38.63 ± 5.23			2.4 ± 1.4	1.3 ± 0.8
Alanine	2.6 ± 1.3	53.49 ± 11.10	58.78 ± 15.36	76.72 ± 17.16	78.87 ± 17.06		2.1 ± 0.7	2.0 ± 0.8	
Methionine	1.9 ± 0.8	9.57 ± 1.29	11.80 ± 2.26	12.72 ± 2.10	12.75 ± 1.69	1.3 ± 0.7	2.4 ± 0.9	2.2 ± 0.9	
Pyruvate	1.6 ± 0.6	18.00 ± 6.89	9.79 ± 4.28	6.86 ± 2.16	8.63 ± 3.67	1.6 ± 1.5	3.4 ± 1.6	3.1 ± 1.1	
Glutamine	1.5 ± 0.7	56.47 ± 6.29	61.20 ± 8.85	70.91 ± 12.40	73.86 ± 10.25		1.3 ± 0.6	2.1 ± 0.7	1.6 ± 0.8
Citrate	1.8 ± 1.0	12.74 ± 3.82	8.21 ± 3.00	8.26 ± 1.60	8.88 ± 1.77	1.1 ± 0.8	2.5 ± 1.4	1.6 ± 0.8	
Creatinine	3.9 ± 2.5	66.75 ± 27.50	39.57 ± 15.74	34.08 ± 18.03	25.56 ± 4.81	1.5 ± 0.8	1.5 ± 1.0	3.2 ± 1.6	1.1 ± 0.4
GPC + APC	1.6 ± 0.7	23.40 ± 6.93	36.98 ± 11.47	36.68 ± 7.81	38.51 ± 6.45	1.3 ± 0.6	2.5 ± 1.3	3.1 ± 2.1	
Mannitol	1.3 ± 1.0	37.53 ± 9.57	54.32 ± 20.98	43.00 ± 16.08	33.10 ± 4.53				1.1 ± 0.4
myo-Inositol	2.0 ± 1.0	15.17 ± 5.50	11.17 ± 5.00	8.65 ± 3.47	5.89 ± 2.22		1.4 ± 0.8	3.3 ± 1.3	1.4 ± 0.7
Lactate	10.8 ± 26.8	152.16 ± 49.37	95.88 ± 24.85	121.44 ± 36.64	145.22 ± 36.47	1.5 ± 1.0			1.4 ± 0.7
Glucose	3.2 ± 5.3	63.80 ± 30.13	100.86 ± 41.23	109.42 ± 37.96	101.70 ± 21.66	1.1 ± 0.6	1.4 ± 0.6	1.8 ± 1.0	
Histidine	1.0 ± 0.5	2.55 ± 0.96	0.78 ± 1.12	0.70 ± 1.12	0.92 ± 1.50	3.0 ± 1.7	2.3 ± 1.3	1.7 ± 1.4	
Hippurate	2.2 ± 0.9	14.43 ± 8.88	3.03 ± 2.31	3.11 ± 0.62	3.88 ± 1.04	1.6 ± 0.8	1.8 ± 0.6	1.6 ± 0.5	
Adenine	1.6 ± 1.0	4.56 ± 3.60	1.04 ± 0.39	1.16 ± 0.41	1.44 ± 0.48	1.1 ± 0.4			
Leucine		30.06 ± 6.39	34.07 ± 5.46	31.74 ± 5.52	36.02 ± 4.48			1.0 ± 0.8	
3-Metyl-2-oxovaleratte		4.18 ± 0.69	3.31 ± 0.60	3.50 ± 1.11	4.38 ± 0.72	2.1 ± 1.7			2.0 ± 1.0
Choline		17.65 ± 4.25	12.59 ± 3.01	13.42 ± 3.04	16.38 ± 2.79	1.4 ± 0.8			1.1 ± 0.8
Tyrosine		5.39 ± 0.99	7.21 ± 2.19	7.72 ± 2.22	8.13 ± 2.02	1.0 ± 0.6	1.3 ± 0.7	2.1 ± 1.1	
3-Hydroxy isobutyrate		3.66 ± 0.95	4.54 ± 1.47	3.95 ± 1.41	3.40 ± 0.75				1.1 ± 0.7
Acetone		4.10 ± 2.27	5.41 ± 2.67	4.29 ± 2.21	3.24 ± 0.89				1.1 ± 0.4
Propylene glycol		3.88 ± 0.89	3.52 ± 0.48	3.72 ± 1.03	4.48 ± 0.88				1.1 ± 0.5
Succinate		3.54 ± 1.71	6.29 ± 3.50	7.00 ± 2.93	4.90 ± 2.50		1.5 ± 0.8		
Betaine		11.46 ± 2.87	13.48 ± 2.17	14.40 ± 2.21	12.48 ± 2.35		1.7 ± 1.1		

Bootstrapping was used to estimate the mean selectivity ratio (SR) ± standard deviation (SD) for each metabolite. The mean SR values ± SD of the pairwise models are only listed for those metabolites that had P-values lower than 0.05.

Looking at the projections presented in Fig. 1b,d, it can be pointed out that after transplantation the individual patients had relatively higher levels of valine, alanine, glutamine, methionine, GPC + APC, mannitol, glucose and lower levels of creatinine, citrate, myo-inositol, lactate, histidine, hippurate and adenine compared to the metabolite levels that were determined before transplantation. These changes are also indicated by the mean content values that are listed in Table 1. Although there was a clear distinction among the samples of ‘T1’, ‘T2’ and ‘T3’ in Fig. 1a, the projections of the same samples that were represented by a reduced number of metabolites, overlap somewhat in Fig. 1d. This may be explained by the fact that the individual metabolic differences over these specific time points after transplantation were not explained well by the metabolites that were selected using ANOVA-TP. This was also confirmed by the low percentages of the sensitivities for the samples of ‘T1’, ‘T2’ and ‘T3’. Once again there was a very clear distinction between the ‘before’ and ‘after’ metabolic states. The sensitivity and specificity of the models with selected metabolites are presented in Supplementary Table S1 in the supplementary material.

**Figure 2:**
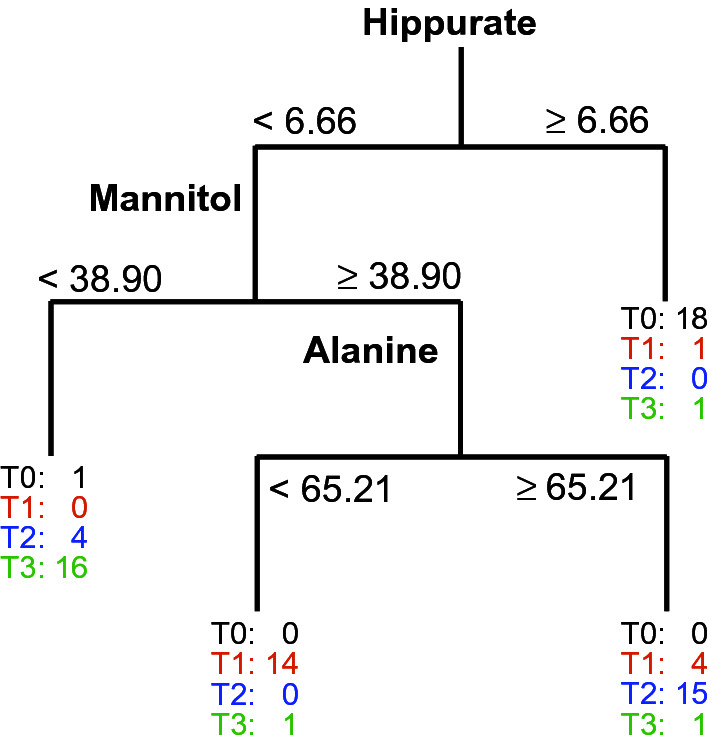
Classification tree that was constructed for 76 blood samples collected from 19 individuals with a target variable that described all four time points. The tree was grown using the ‘time’ factor matrix that was obtained from ANOVA summed with the residual matrix (ANOVA-CART).

As was mentioned earlier, we were also interested in finding a small number of metabolites using CART, the binary splits of which at given threshold values are responsible for the metabolic changes in individuals over time. The binary tree for the ANOVA ‘time’ factor matrix summed with the residual matrix (the same matrix as in ANOVA-TP) using the categorical response variable, which is associated with ‘time’, is presented in Fig. 2. This methodology combines ANOVA and CART and hereafter, it will be referred to as ANOVA-CART.

An important observation, which can be observed in Fig. 2, is that hippurate was the metabolite that enabled the samples that had been collected before transplantation to be distinguished with fairly high sensitivities of 94.7% (ANOVA-CART, Supplementary Table S1) and high specificities of 96.5%. Higher values of hippurate were also characteristic for the individuals before transplantation which was in agreement with the results from the ANOVA-TP. However, for ANOVA-CART, only one metabolite was used for this discrimination and the threshold value for this metabolite was identified. Values lower than the threshold values for hippurate (< 6.66) were found for the majority of individuals after transplantation in ANOVA-CART. Please note that the data are PQN-transformed and only the ‘time’ variation was modeled. Values of mannitol that were lower than 38.90 (Fig. 2, Table 1) were characteristic for the majority of individuals who were six months after renal transplantation (‘T3’). Compared to the results from the ANOVA-TP, ANOVA-CART offers an enhanced sensitivity and a comparable specificity (see Supplementary Table S1 in the supplementary material). Considering the larger number of metabolites (19 metabolites) that were selected in ANOVA-TP, ANOVA-CART is to be preferred in this case. The most difficult distinction was for the metabolic state in the intermediate period after transplantation (T1 and T2). Relatively poor sensitivity values were obtained from the ANOVA-TP, even though fairly high specificities were found. Better sensitivities and fairly high specificities were found for the ANOVA-CART when the binary split was performed on alanine. From this comparison, one can conclude that three metabolites such as hippurate, mannitol and alanine may be enough to characterize the metabolic changes over time with good sensitivities and specificities. It should be noted that these results are interpreted more in an explorative context here and are not being used solely to interpret the performance of the various models.

**Figure 3:**
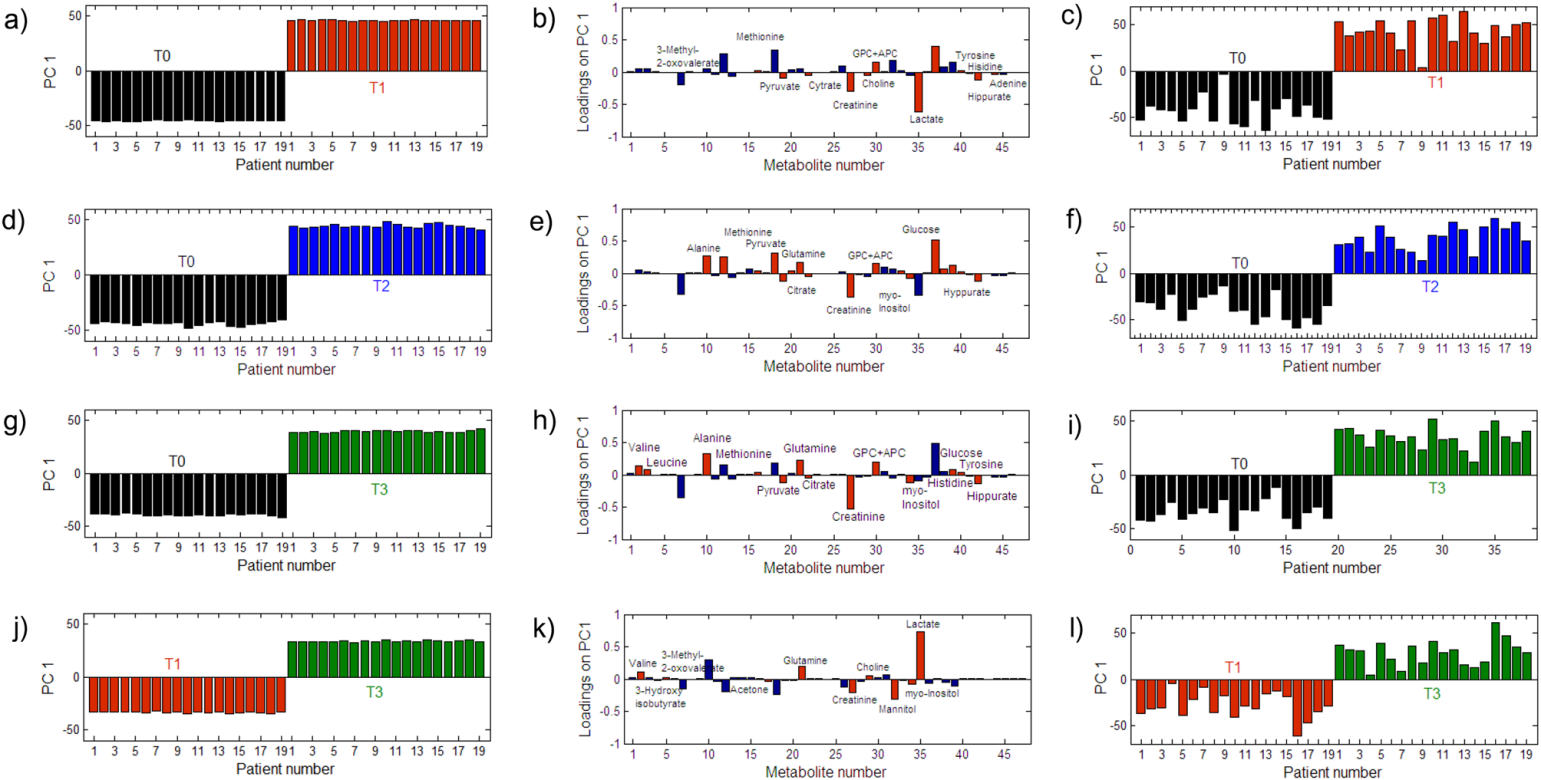
Target projection scores (**a**,**d**,**g**,**j**) and loadings (**b**,**e**,**h**,**k**) vector that were associated with the ‘time’ effect from the ANOVA-TP with all of the metabolites as well as the target projection scores (**c**,**f**,**i**, **l**) from the ANOVA-TP with selected metabolites (Table 1) for T0 vs. T1 (**a**–**c**), T0 vs. T2 (**d**–**f**), T0 vs. T3 (**g**–**i**) and T1 vs. T3 (**j**–**l**).

Another way to characterize the metabolic changes is to look at the pairwise-group comparisons. For example, it might be interesting to find which metabolites best characterize the ‘before’ and ‘after’ (T0 vs. T1 or T0 vs. T2) transplantation conditions, e.g. which metabolites are associated with the changes after a relatively long period of time following the transplantation (T0 vs. T3) or that characterize only the changes in the post-transplantation period (T1 vs. T3), and furthermore, to possibly answer the question of whether the levels of the same metabolites are expected to be perturbed in all of these cases. The results of the pair-wise comparisons that were obtained from ANOVA-TP are presented in Fig. 3. The ANOVA-TP for any of the two-group analyses produced one target projection loadings vector and one scores vector. Once again, the bootstrapping procedure was used to estimate the average value of the selectivity ratio for each metabolite. The cut-off value for the selectivity ratio was determined to be 1.0. The metabolites that were found to be important are presented in Table 1. The *P*-value of each metabolite (its loading value) was also calculated using the bootstrapping procedure.

The results from ANOVA-TP showed a clear distinction between any two groups of the samples that were represented by all of the variables using only one latent variable due to the better description of the within-group variation. Again, a decrease in the levels of creatinine, hippurate, lactate, adenine, citrate, choline, pyruvate and GPC + APC was observed along with increasing levels of glucose and tyrosine in the individuals after transplantation (Fig. 3a). A similar tendency was observed for the longer period of observation (T0 vs. T3).

**Figure 4:**
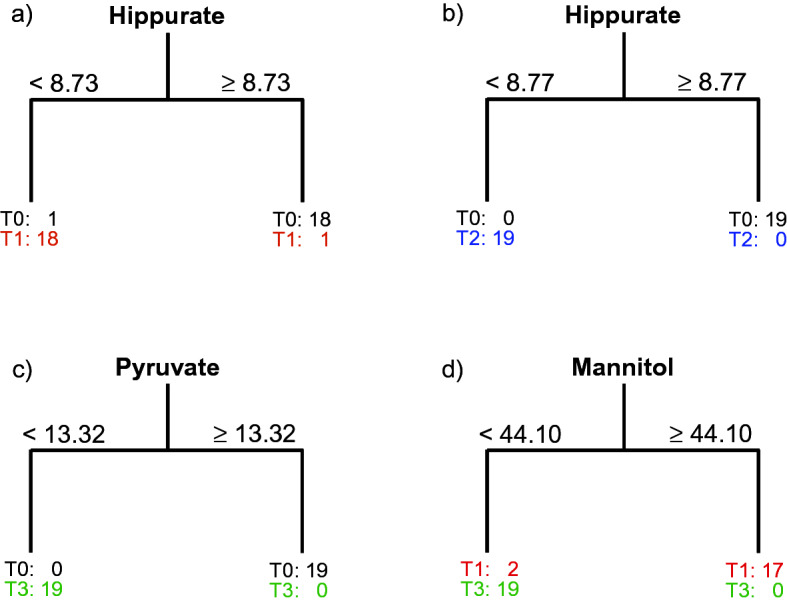
Classification trees that were obtained from the ANOVA-CART for the blood samples that had been collected from 19 individuals with the target variable describing (**a**) the ‘before’ and ‘after’ transplantation samples (T0 vs. T1), (**b**) T0 vs. T2, (**c**) T0 vs T3 and the period after transplantation (**d**) T1 vs. T3.

Additional metabolites that were found to be important were valine, alanine, leucine, and glutamine, the levels of which increased during this period of time, while the level of myo-inositol decreased. The results of all of the before-after models not only indicated a decrease in the creatinine level, which was an expected observation, but also some important changes in the levels of other metabolites. If one investigates the changes only after transplantation (model T1 vs. T3), several characteristic metabolites such as mannitol, 3-hydroxy-isobutyrate, acetone and propylene glycol, the levels of which appear to decrease with the time after the kidney intervention, are considered to be important. The pairwise ANOVA-CART models are presented in Fig. 4, while the pairwise CART models are presented in Supplementary Fig. S3 in the supplementary material.

Trees that are presented in Fig. 4 and the values of the sensitivities and specificities that are listed in Supplementary Table S1 confirm the results from the four-group models. Once again, only one metabolite, hippurate, was enough to distinguish between the samples from the same individuals who were ‘before’ and ‘after’ transplantation (T0 vs. T1). Values lower than 8.73 for the ANOVA-CART model were characteristic for the samples after renal transplantation (T1). Compared to the ANOVA-TP, only one variable was needed with the ANOVA-CART, which gave a sensitivity and specificity of 94.7%, to describe T0 vs. T1 changes. A surrogate variable, the split on which gave the same ANOVA-CART performance, was histidine. All of the patients who were after transplantation also had levels of histidine that were lower than 1.67. When investigating the longer before-after period (T0 vs. T2), the same performance in terms of sensitivities and specificities was obtained using ANOVA-TP and ANOVA-CART, but once again only one variable (hippurate) was used in ANOVA-CART (Fig. 4b) and CART (Supplementary Fig. S3). What is important to emphasize here is that the levels of hippurate in almost all of the patients with the exception of one patient were lower than 8.73 1 day after the transplantation, but all of the patients had hippurate levels lower than 8.77 after seven to ten days post-transplant. Six months after renal transplantation, all of the patients had a level of pyruvate that was lower than 13.32 with respect to its level before transplantation (Fig. 4c). This was indicated by ANOVA-CART from the data for the longest before-after period (T0 vs. T3). Both methods, ANOVA-TP and ANOVA-CART, had the same best performance (Supplementary Table S1). A surrogate variable in ANOVA-CART was tyrosine, however, its level changed differently than pyruvate. Six months after transplantation, all of the patients had tyrosine levels that were higher than 6.76, although it was lower before transplantation. Analysis of the results from CART for the PQN data showed that after transplantation, all of the patients had levels of creatinine that were lower than 37.51 (Supplementary Fig. S3 in the supplementary material), while only two of them had such a low level of creatinine before transplantation. This analysis suggests that the hippurate level could be useful for investigating the short and longer (up to 7–10 days) periods of changes after transplantation, while the changes in the level of pyruvate seem to be more sensitive than the changes in the level of creatinine in a much longer term (up to 6 months) of ‘before’ and ‘after’ monitoring. Finally, if one is interested in only monitoring the period after transplantation, it seems that the changes of mannitol are enough to obtain a good sensitivity and a specificity of 100%. A level of mannitol that was lower than 44.10 was characteristic for all of the individuals that were up to six months after transplantation, while a higher level of mannitol was only found in 17 of them one day after transplantation (Fig. 4d).

### Investigating the inter-individual metabolic changes

**Figure 5:**
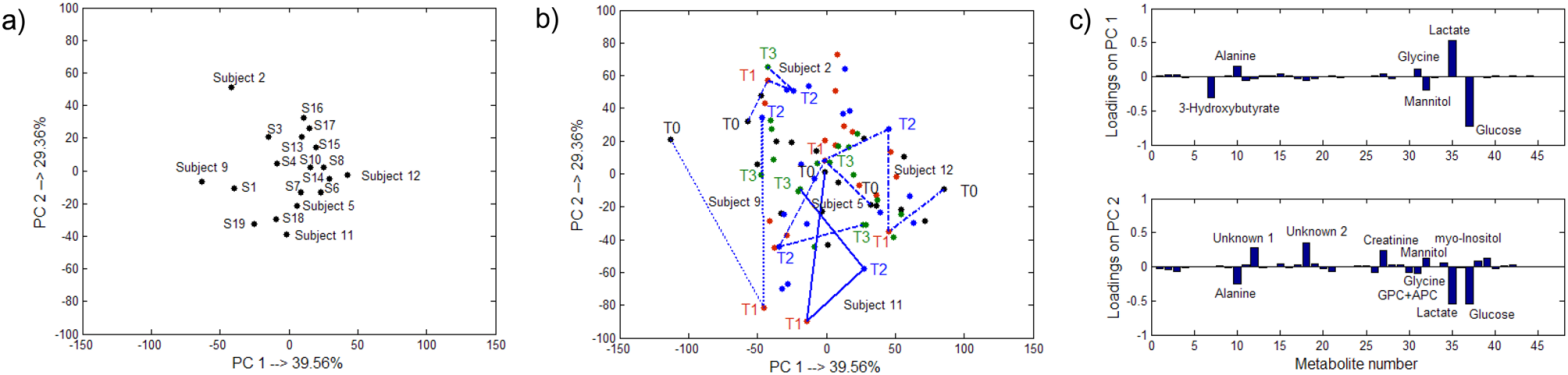
Results obtained from ASCA for the inter-individual changes: (**a**) the centroids of the individuals over time that were projected in the space that was spanned by PC1 and PC2, (**b**) the trajectories for some selected individuals in the space spanned by PC1 and PC2 and (**c**) projections of the metabolites on the PC 1 and PC2.

In order to investigate the inter-individual metabolic changes, the ASCA method was used, because these differences seemed to be more pronounced. The individual trajectories only will be discussed for exploratory purposes. The results are presented in Fig. 5.

Even though the general tendencies for the metabolite changes over time were similar for the majority of individuals, some individuals may have different trajectories of changes. As was mentioned earlier, only the patients with the serum profiles that are associated with normal recovery were considered in our study. Figure 5a from the ASCA indicates several such subjects/individuals. These subjects were nos. 2, 9 and 12. Their trajectories, which are presented in Fig. 5b, mainly indicated changes in glucose, lactate, alanine, creatinine and mannitol (with high absolute loading values). These metabolites were also found important with ANOVA-TP. Subject no. 9 showed different tendency of changes in levels of the lactate and creatinine compared to the majority of individuals. Compared to time T1, the level of lactate was lower before (T0) transplantation, while the level of creatinine was higher after (T1) transplantation compared to the sample that was collected before graft intervention. An increase in the level of glucose is also observed. The level of glucose was highest among all subjects. The levels of creatinine and mannitol increased further over time (T2), while the level of lactate and glucose decreased. These changes indicate a delayed graft function. Furthermore, the trajectory suggests a drop in the creatinine and mannitol levels (T2), while the level of lactate increased. The trajectory of subject no. 12 suggests oscillation-like changes of glucose and lactate. Compared to the levels of glucose and lactate before transplantation (T0), the level of glucose was higher, while the level of lactate was lower after transplantation (T1). Then (at T2), the level of glucose decreased and the level of lactate increased, but not as low or high as the initial values. Finally, the level of lactate decreased again, while the level of glucose increased. The level of lactate was the highest.

Similar changes of the lactate and glucose levels were observed for individual no. 2, but the level of lactate was the lowest and the magnitude of changes was much smaller as the trajectory suggests.

## Discussion

### Interpreting the changes in the metabolite levels over time

**Figure 6:**
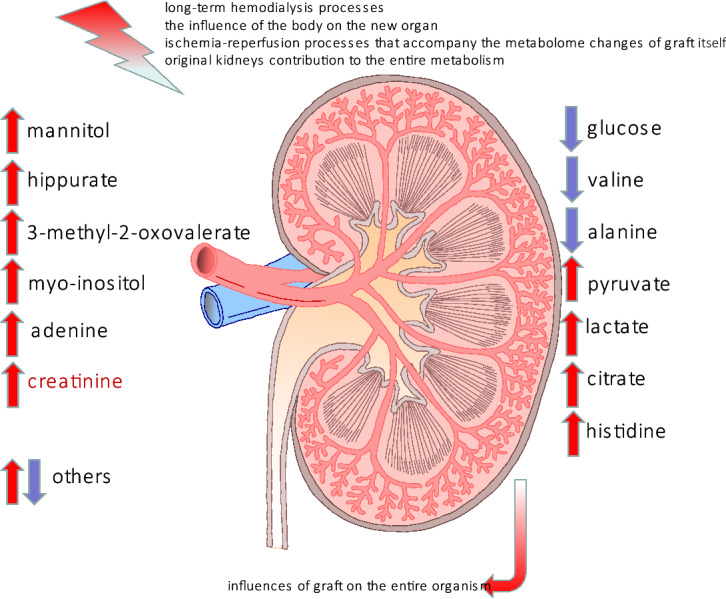
Schematic representation of the external and internal stimuli that influenced the changes in the serum metabolites at T0. The up-regulated metabolites are represented by red arrows while the down-regulated metabolites are indicated by blue arrows.

The course of metabolite changes may be characterized by either consecutive increases or decreases or by specific metabolite perturbations between specific intervals of time. Some specific metabolite changes were more pronounced when investigating the inter-individual changes. The most important and significant change in metabolite levels was observed as was expected ‘before’ and ‘after’ transplantation (T0 and T1) as well as at the beginning and at the end of the adaptation period (T1 and T3). The first monitoring time point after transplantation, T1, is considered to be the metastable state, in which all of the processes are connected, while the second monitoring time point, T2, is associated with the transition stage in which the recovery processes are at different levels of completeness. The duration of the adaptation stage very often depends on the graft conditions (age, donor lifestyle etc.), but in clinical practice, the adaptation period is supposed to be completed ca. six months after the renal surgery. In general, the molecular content of serum can vary over time for at least four reasons: (i) long-term hemodialysis processes, (ii) functioning of the new graft that affects the entire organism, (iii) the influence of the body on the new organ and (iv) the ischemia–reperfusion processes that accompany the metabolome changes of the graft itself. The variation of selected serum metabolites is illustrated schematically in Fig. 6. It is very important to emphasize that the kidney that is not functioning is usually left in the body and its effect on the entire organism is still unknown.

The results from the ANOVA-TP confirmed the importance of monitoring the creatinine level ’before’ and ‘after’ transplantation as an indictor of renal transplant function. The results are also in agreement with some previous observations that creatinine is a long-term post-transplantation indicator, whose level becomes abnormal in the later stages of kidney dysfunction^18^. The lack of a decrease or even an increase in creatinine level as in subject no. 9 in our study may be explained by a delayed graft function.

An interesting finding here is that hippurate had an increased level before renal transplantation, which then decreased throughout the post-operative period. Hippuric acid, a recognised uremic toxin that is associated with the gut microbiome^29^ can be cleared during hemodialysis^30^. The main route of its elimination is via an active renal tubular secretion^31^ and its dysregulation can be the main reason for its accumulation in the blood^32^. Furthermore, hippurate was found to be an inhibiting agent for glucose utilization in the muscles^33^ and an endogenous stimulator of ammoniagenesis^34^. A high level of hippurate is accompanied with a lower level of glucose before transplantation. The reason for a low glucose level may be that the blood samples were collected from fasting patients. However, the increase in the glucose level after transplantation (T1) can be explained by a short-term impaired glucose metabolism. Moreover, after graft transplantation, patients are treated with calcineurin inhibitors and steroids which also increase the glucose level in blood and may lead to insulin resistance and diabetes in long-term treatment^35–37^. In general, during the adaptation period (T2–T3), the glucose level decreased and stabilized. Subject no. 12 experienced a lack of stability in the glucose and lactate levels over time, which may be explained by the influence of post-transplantation therapy or by a hypoxia/ischemia condition in which the level of lactate changes along with the level of pyruvate.

Changes in the glucose level can also be related to changes in the level of valine. As a branched chain amino acid (BCAA), valine can participate in glucose metabolism as glucogenic amino acid. Its catabolism was found to be preferential for uremic patients^38^. The increasing trend in the levels of valine in our study is partially in agreement with the tendency presented in literature (e.g. valine is not statistically important)^39^. Furthermore, the changes in the glucose level were very similar to those that were observed for glutamine and this can be associated with the initiation of the TCA cycle. The lowest level of glutamine was observed before transplantation (T0), which is in contradiction to the fact that a high level of glutamine is associated with a low glomerular filtration rate, GFR, (21–39 ml/min)^39^. Several animal translation studies have demonstrated that the administration of glutamine ameliorates inflammation after an ischemia–reperfusion injury in rats^40^. Moreover, the level of glutamine decreases in the post-transplantation period (T1–T3).

The higher level of alanine during the adaptation period compared to its level before transplantation can be reversibly associated with the levels of pyruvate. In fact, one of these metabolites can be the substrate for the other. Another interesting fact is that the ratio of Ala/Gln at all of the time points was close to 1 [from 0.94 (T0) to 1.08 (T3)].

The restart of graft functioning is associated with the level of GPC + APC. After renal surgery, its level is the highest (T1) and normalizes by the end of the adaptation period (T3). These changes can be explained by the breakdown of lipids, which is associated with a membrane breakdown and its remodeling activity^41^. An opposite tendency has also been reported in the literature in which a decreased level of GPC was recorded after a sham operation, which simulated a renal ischemia–reperfusion injury (IRI) in mice, although its level normalized during the adaptation period^41^.

In our study, the level of methionine increased after transplantation and then its level decreased. This has been found to be beneficial for the kidneys^42^. In general, a low level of methionine is characteristic for chronic kidney diseases^43^, but this is not associated at the baseline GFR. However, in another literature source^44^, it was shown that patients with end-stage renal disease had normal levels of methionine and betaine. The latter metabolite fluctuated from a lower to higher level, and was at its lowest level in T3.

Mannitol is a sugar alcohol that was not found in a healthy human serum metabolome^45^ using NMR spectroscopy measurements. Unfortunately, we determined its presence in the serum samples that were collected before (T0) and 6 months (T3) after transplantation. Its presence and role are not very clear and it would be a bold assumption to state that mannitol is associated with kidney injury and renal diseases^46^. Even though the highest level of mannitol (T1) and myo-inositol, which is another polyol, can be strongly associated with renal failure before transplantation, their concentrations decreased in the period of recovery.

Among the metabolites whose content decreased over time were pyruvate, citrate, lactate, histidine and adenine. A surprising observation was that the levels of pyruvate and citrate, which are considered to be TCA fuel, were higher before transplantation. This might reflect either accelerated glycolysis processes that are shifted towards producing pyruvate or with a decreased activity of the TCA enzymes, which is bottleneck that blocks the utilization of citrate. However, the fluctuation in the level of succinate may only partially support this hypothesis. The results of a comparative study that was carried out showed that there were no differences between a group of patients with a long-term hemodialysis who underwent citrate infusions and a control group^47^. Another possible mechanism of citrate and pyruvate accumulation can be associated with the hypoxia/ischemia condition^48^ prior to transplantation in which an increased level of lactate is observed. The highest level of lactate can also be associated with its disrupted disposal and uptake by native kidney^49^. Furthermore, lactate is a product of the LDH transformation of pyruvate: LDH was found to be associated with tubular injury during the short-term post-transplantation period that concerned ischemic ATN (acute tubular necrosis)^50^. Lactate also has an increased level in patients before hemodialysis^51^.

Changes in the histidine level^52^ are closely related to changes in the level of glutamate. This amino acid has a protective role as an anti-inflammatory agent because it is a scavenger of reactive oxygen species^53,54^. A decreased level of histidine in urine can indicate an injury to kidney function^39,53^, while a low level of histidine in the serum is characteristic for CKD patients who experience many pathological states^54,55^. In our study, the content of histidine was at its highest level before the kidney replacement therapy, which is not in agreement with the findings that have been reported in the literature. The second aromatic amino acid, tyrosine, was down regulated before renal transplantation, which could have been associated with the renal biosynthesis of tyrosine in the kidneys^55^, where after the incorporation of graft functioning, the tyrosine level was stabilized in T2 and T3. A similar behavior was also found for the choline level, where the level of this metabolite was higher before than after graft transplantation, which is in agreement with the literature data^56^.

The highest level of adenine before transplantation, which is an indication of renal failure, is in a good agreement with the results reported in the literature^57–59^. Adenine-induced CKD is a frequently used model for investigating different effects on renal distortion. It was shown experimentally that a long-term feeding of rats with adenine may damage the tubules and glomeruli^60^. An excess of adenine can be utilized by xanthine dehydrogenase to 2,8-dihydroxyadenine (DHA)^61^,which because of its low solubility precipitates in kidney tubules^61,62^. In our study, the excess of adenine in the serum at T0, which was triggered by the renal transplant, was equalized over time by its utilisation to other products^62,63^.

When investigating the fluctuations in the concentrations of the ketobodies, it was observed that 3-hydroxybutyrate and acetone were at the highest level in T1, and that their level had decreased by T3. 3-hydroxybutyrate can be converted into acetoacetate, which can spontaneously decarboxylate into acetone^64^. The highest level before transplantation can be associated with a lower level of glucose and the ketogenesis processes. 3-methyl-2-oxovaleric acid, which is a ketoanalogue of isoleucine, is a neurotoxin, an acidogen, and a metabotoxin^65^. However, recently, the calcium salt of this compound has been used as an ingredient in supplements that ameliorate the risk of a long-term dialysis^66^. The level of this ketoacid fluctuated in our study.

Although propylene glycol is routinely found in the blood serum at a concentration of 22 μM, it is recognised as an “external concomitant” that originates from the cosmetics, food and toothpaste that an individual has used^42^. Its level was higher before graft transplantation and then was maintained at a constant level.

All of these metabolite changes over time are described for the patients who had progressed to better health in a six-month period, however, it should also be considered that the unknown activity of a native kidney can overlap with the proper functioning of a graft.

## Conclusions

In this study, we identified the concentration changes of metabolites that can be used to describe the recovery period towards a better health condition after graft transplantation surgery. This was done using the repeated measures methodology in ANOVA-TP and ANOVA-CART. The ANOVA-CART is proposed here under the assumption that a small set of metabolites the orthogonal splits on which may explain the metabolic differences over time better in some cases than a linear combination of the metabolites. The capability of this method was justified by the relatively high sensitivities and specificities that were obtained for all of the models. The minimum number of metabolites that can be used to monitor renal function includes hippurate, mannitol and alanine. Specifically, it was found that the level of hippurate was more sensitive to the changes in renal function, while monitoring the creatinine is appropriate for indicating large changes in renal function such as those before/after graft surgery. This is a very promising result that needs to be investigated further. It will be interesting to determine whether hippurate can be suitable as an additional indicator for a kidney biopsy in the early stages of kidney injury. A general unrecognized problem is that the native kidney which is usually not removed during the transplantation surgery, may influence the general metabolic picture. Since the renal allografts change the metabolic patters dynamically over time, it is not surprising that some metabolites become important only at a certain time after renal transplantation, thereby reflecting the molecular processes that are characteristic for the recovery of health.

## Materials and methods

All experiments were performed in accordance with the Polish legal laws and regulations and the experimental protocol was approved by the Bioethical Committee of Wroclaw Medical University (KB148/2013).

The clinical characteristics of the patients are presented in Table 2.

**Table 2:** Demographic and clinical parameters of patients in the study.

Characteristics				
Recipient age in years (mean ± SD)	55.5 ± 13.6			
Male gender, no (in %)	13 (68%)			
Female gender, no (in %)	6 (32%)			
**Cause of chronic renal failure no.**				
Chronic glomerutonephritis	6			
Diabetic nephropathy	2			
Hypertonic nephropathy	3			
Interstitial nephritis	3			
Polycystic kidney disease	2			
Other	3			
**No. of all of the presensitized patients**	8			
PRA < 10%	4			
PRA 10–50%	3			
PRA > 50%	1			
No. of HLA mismatches (mean ± SD)	4.6 ± 1.0			
Donor age in years (mean ± SD)	46.3 ± 16.3			
CIT (mean ± SD)	25.3 ± 4.6			
Loss of the graft during 12 months	None			
Patients with proteinuria in the 12 month	None			
Characteristics of hypertension	Before transplantation	Three months	Six months	12 months
RR	132/80	132/83	139/80	137/79
No. of antihypertensive drugs	1.5 ± 1.4	1.5 ± 0.8	1.3 ± 0.8	1.6 ± 1.0
Creatinine (mg dL^−1^)		1.58 ± 0.40	1.49 ± 0.31	1.45 ± 0.35

*PRA* panem reactive antibodies, *HLA* human leukocyte antigen, *CIT* cold ischemia time.

### Sample collection

Peripheral venous blood samples were drawn from all of the participants after overnight fasting for at least 8hours. Blood samples were collected using Sarstedt S-Monovette system serum tubes (Sarstedt AG & Co., Nümbrecht, Germany), which were centrifuged at 1000 × *g* for 15 min at 4 °C. The serum samples were stored in 1.5 ml Eppendorf safe lock tubes and maintained at − 80 °C until the analysis. The study group included 19 patients whose levels were monitored over time [defined as time 1 (1 day), 2 (7–10 days) or 3 (6 months)] after transplantation and were interpreted with respect to their levels before transplantation (defined as ‘T0’).

All experiments were performed in accordance with the Bioethical Committee of Wroclaw Medical University (KB148/2013): each subject signed a written informed consent.

### Sample preparation for proton NMR spectroscopy

The serum samples that were collected were thawed at room temperature and vortexed. Then, 300 μl of the serum was transferred to a new Eppendorf tube and mixed with 700 μl of cold methanol for protein precipitation. Next, the samples were homogenized (Qiagen, Tissuelyser LT) for 10 min at 50 Hz and then incubated for 20 min at − 20 °C. The homogenization step and incubation were then repeated. Subsequently, the mixtures of serum-methanol were centrifuged for 30 min at 15,000 rpm at 4 °C. An aliquot of 700 μl of supernatant was transferred to a new Eppendorf tube and then evaporated to dryness in a vacuum centrifuge (JWElectronic WP-03) for 5 h at 1,500 rpm at 40 °C. The dry precipitate was dissolved in 600 μl PBS (0.5 M, pH = 7.2, 20% D_2_O, and 330 μM TSP) and then 550 μl of the mixture was transferred to an NMR tube (SP, 5 mm ARMAR Chemicals). The samples were maintained at 4 °C before being measured. The same serum sample preparation and ^1^H NMR measurement protocols were used in our previous study^67^.

### ^1^H NMR measurements

The NMR spectra of serum were recorded at 300 K using an Avance III spectrometer (Bruker, GmBH, Germany) operating at a proton frequency of 600.58 MHz. The NMR spectra were recorded using a *cpmg1dpr* pulse sequence with water presaturation in a Bruker notation. For each sample, 128 continuous scans were collected with a spin-echo delay of 400 μs; 80 loops; a relaxation delay of 3.5 s; an acquisition time of 2.73 s; a time domain of 64 k and a spectral width of 20.01 ppm. The 1D spectra were processed with a line broadening of 0.3 Hz, manually phased, baseline-corrected using MestReNova software (Mestrelab Research v11.0) and referenced to the TSP signal δ = 0.0 ppm. The methanol and water resonance signals were removed from the data matrix. All of the spectra were normalized to the TSP resonance signal. The alignment of the resonance signals was completed using a correlation optimized warping algorithm (COW) and the icoshift algorithm, which was implemented in MATLAB (v R2014a, Mathworks Inc.)^67^.

### Methods for data analysis

Since the levels of 47 metabolites were measured for the same patients over time, methods that take into account the repeated measures structure of data were used for the analyses in this study. The core of all of these methods is to collect factor mean estimations for the metabolites in respective factor matrices using the specific analysis of variance, ANOVA, principal and to explore or model the variance of the collected factor matrices using principal component analysis, PCA; simultaneous components analysis, SCA; partial least squares-discriminant analysis, PLS-DA or PLS combined with the target projection method, TP. With the repeated measures ANOVA model^68^, the total variability of each metabolite is partitioned into the measurement variability that is attributable to the different time points, which is referred to as SS_time_, the variability due to individual differences among patients (SS_patients_) and the individual variability at each time point, which can be denoted as SS_error_. Thus, the respective factor matrices will be of the same sizes 76 (19 *individuals* × 4 *time points*) × 47 *metabolites* and will represent the effects of ‘time’ and ‘patients’, while the residual matrix will contain the individual measurement errors. The multivariate analysis is carried out by the different methods. ANOVA-PCA^24^ uses principal component analysis on any factor matrix summed with the residual matrix in order to describe the within-group variance better, while simultaneous component analysis is used for the direct analysis of the factor matrices, which is the essence of the ASCA method^25^. A further improvement of the description of the within-group variance can be obtained from the analysis of variance-target projection approach, ANOVA-TP^27^. For the multi-group problem that is studied here, the PLS2 variant enabled working with several coded response variables. Because of the closure effect, the four groups in these data (associated with the four time measurement points) could be modeled with PLS2-DA using three response variables that were coded by − 1 and 1. The model complexity was estimated using the leave-one sample-out approach. Once a vector of regression coefficients was obtained for each of the responses, the target projection approach was performed. The result is a target projection matrix which has a rank that equals the number of groups minus one. The mean and uncertainty of the selectivity ratio^23^, SR, value for each metabolite were determined using a bootstrapping procedure with a block replacement. This meant that each of the 10,000 bootstrap samples contained the metabolite measurements over time for the individuals that were selected randomly with a replacement (a total of 19 individuals). A unitary cut-off value for the selectivity ratios was used here. This selection was made after using the discriminating variable test, e.g. the DIVA test^69^. The *P*-value for each metabolite was also estimated using a bootstrapping of the loadings after the target transformation. Only *P*-values lower than 0.05 were considered in the interpretation.

The goal of CART is to find some explanatory metabolites for which the binary splits at a suitable threshold value form as homogeneous groups of samples as possible. The results are visualized as a binary tree, which consists of a number of nodes that represent the subgroups of the data samples. For example, Fig. 4a presents a simple binary tree with two terminal nodes. Terminal nodes are the nodes that cannot be split any further. The optimal split was obtained at a hippurate value of 8.73 (mean value of a given variable for two neighboring samples from the two groups, which led to the largest increase in homogeneity after the binary split). In order to obtain the optimal binary splits, entropy was minimized as an impurity function. The most homogeneous nodes, e.g. the pure nodes that were obtained from the split, had the lowest value of entropy. The nodes were split while the nodes were not pure. The number of nodes was optimized using the so-called cost-complexity pruning in order to obtain good prediction properties. In our study, binary trees were constructed for the transformed data and for the respective ‘time’ factor matrix summed with the residual matrix, which were obtained from the multiple ANOVA analyses. The independent variable represented the respective time points.

All of the calculations were performed on a personal computer (Intel(R) Pentium(R) M, 1.60 GHz with 2 GB RAM) using Microsoft Windows XP (service pack 2) operating system using in-house programmed algorithms and Statistical Toolbox 8.0 with MATLAB 7.0 (R14).

### Ethics approval and consent to participate

The study protocol was approved by the Bioethical Committee of Wroclaw Medical University (KB148/2013): each subject signed a written informed consent. We definitely and unequivocally declare that the research that was described in our manuscript did not involve, in any manner, organs procured from prisoners. All transplanted organs were retrieved via the transplant team of the Department of Vascular, General and Transplantation Surgery of Wroclaw Medical University in compliance with all Polish legal laws and regulations that include organ donation possibility only in the case of no objection made by the donor before their death.

## Supplementary information

Supplementary Information.

## Data Availability

The NMR data that were collected and/or analyzed in this study can be obtained from the corresponding author upon request.
