# Supplementary material for: Serum metabolomics approach to monitor the changes in metabolite profiles following renal transplantation
Source: Sci Rep. 2020 Oct 14;10:17223. doi: 10.1038/s41598-020-74245-z (PMC7560840; doi:10.1038/s41598-020-74245-z)
Supplement: Supplementary file 1 — Supplementary Information. [file 41598_2020_74245_MOESM1_ESM.doc]

**Supplementary material**

Title of the article: Serum metabolomics approach to monitor the changes in metabolite profiles following renal transplantation

Authors: Ivana Stanimirova, Mirosław Banasik, Adam Ząbek, Tomasz Dawiskiba, Katarzyna Kościelska-Kasprzak, Wojciech Wojtowicz, Magdalena Krajewska, Dariusz Janczak and Piotr Młynarz

**1. List of the metabolites that were considered in the study**

Isoleucine, Valine, Leucine, 3-Hydroxyisobutyrate, 3-Methyl-2-oxovalerate, Propylene glycol, 3-Hyroxybutyrate, 2-Hydroxyisobutyrate, 2-Phenylpropionate, Alanine, 2-Hydroxybutyrate, Acetate, Proline, NAC, Methionine, Acetone, Pyruvate, Succinate, Glutamine, Citrate, 2-Oxoisocaproate, Dimethylamine, N,N-Dimethylglycine, Creatine, Creatinine, Malonate, Choline, GPC+APC, Glycine, Mannitol, Betaine, myo-Inositol, Lactate, Threonine, Glucose, Tyrosine, Histidine, Hippurate, Adenine, Inosine, Formate, Trigonelline and five unidentified metabolites.

**2. Analysis of data using PCA, ANOVA-PCA and ASCA**

PCA was carried out on the PQN-normalized and centered NMR data. The projections of all 76 samples on the first two principal components are presented in Fig. S1a. Although there was not a clear grouping tendency that might suggest metabolic changes over time, it was observed that the individual metabolic profiles varied more before transplantation (T0) than the individual profiles after transplantation (T1, T2 or T3). Figs. S1b and c illustrate the differences between the ANOVA-PCA and ASCA methods for the ‘time’ effect. Compared to the results of the PCA, there was a clearer difference between the metabolic profiles of the patients before and those after the kidney transplantation. Similar to the PCA results, it was not possible to make a clear distinction among the groups of samples that were collected over the entire post-transplantation period.

Fig. S1 Projection of the samples in the space spanned by the first two latent components that were obtained from a) PCA, b) ANOVA-PCA and c) ASCA along with their 90% confidence ellipses, which were placed at the centroids of the groups that were defined according to the ‘time’ factor

**3. Analysis of the data using CART**.

CART was conducted on the PQN normalized data. The tree that was grown using a response variable that described the ‘time’ of sample collection is presented in Fig. S2. Similar to the results of ANOVA-TP and ANOVA-CART that are presented in the main text of this article, hippurate was the metabolite that was responsible for distinguishing the ‘before’ and ‘after’ transplantation samples. Levels of hippurate that were lower than 5.88 were characteristic for the majority of the samples after transplantation. The samples that were collected from the individuals who were six months after renal transplantation (‘T3’) also had levels of 3-methyl-2-oxovalerate that were higher than 4.27 (Fig. S2). Once again, similar to the results of ANOVA-TP, it was difficult to distinguish between the samples that were collected in the intermediate period after renal transplantation (‘T1’ and ‘T2’), which was also indicated by the relatively low percentages of sensitivities and specificities in Table S1.

Fig. S2 Classification tree that was grown for the 76 blood samples that had been collected from 19 individuals ‘before’ and ‘after’ renal transplantation with a target variable that described all four time points. The tree was grown using the original PQN-transformed data.

Classification trees for the pairwise-group comparisons that are associated with the ‘before’ and ‘after’ (T0 *vs*. T1, T0 *vs*. T2 or (T0 *vs*. T3) transplantation conditions or that characterized only the metabolic changes in the post-transplantation period (T1 *vs*. T3) are presented in Fig. S3. Similar to the results of ANOVA-CART, CART also identified hippurate as the metabolite that was responsible for the distinction between the ‘before’ and ‘after’ transplantation samples (T0 *vs*. T1 and T0 *vs*. T2). All of the patients had levels of hippurate that were higher than 3.28 before transplantation, which indicates a sensitivity of 100%, while 16 out of 19 patients had lower levels of hippurate one day after the renal intervention, which resulted in a specificity of 84.2% (Fig. S3a and Table S1). Ten days after the renal intervention, all of the patients had levels of hippurate that were lower than 5.25 (Fig. S3b). When the samples that were collected before and six months after renal transplantation were compared, all of the patients had creatinine levels that were lower than 37.51 after the intervention (Fig. S3c). This finding indeed confirmed that the long-term changes in renal function strongly affect the levels of creatinine. Once again, only one metabolite, 3-methyl-2-oxovalerate, distinguished the samples from the post-transplantation period, e.g. T1 *vs*. T3. All of the these pairwise CART models had comparable or lower values of the specificities and sensitivities than those that were obtained from the ANOVA-CART when only the variance of the ‘time’ effect was considered (Table S1).

Fig. S3 Classification trees that were constructed for the blood samples collected from 19 individuals with the target variable describing before’ and ‘after’ transplantation: a) T0 *vs*. T1, b) T0 *vs*. T2, c) T0 *vs*. T3 and d) post-transplantation period, T1 *vs*. T3.

Table S1 Sensitivity (in percent) and Specificity of the models that were built for the metabolites that were selected. The metabolites that were used in the ANOVA-TP are listed in Table 1.

|  | ANOVA-TP | | |  | CART | |  | ANOVA-CART | |
| --- | --- | --- | --- | --- | --- | --- | --- | --- | --- |
|  | (model complexity) | Sensitivity | Specificity |  | Sensitivity | Specificity |  | Sensitivity | Specificity |
| all time points |  |  |  |  |  |  |  |  |  |
| T0 | 7 | 89.5 | 98.2 |  | 89.5 | 98.5 |  | 94.7 | 96.5 |
| T1 |  | 63.2 | 98.2 |  | 47.4 | 100.0 |  | 73.7 | 98.2 |
| T2 |  | 57.9 | 93.0 |  | 94.7 | 71.9 |  | 78.9 | 91.2 |
| T3 |  | 73.7 | 96.5 |  | 68.4 | 96.5 |  | 84.2 | 91.2 |
|  |  |  |  |  |  |  |  |  |  |
| pairwise  comparisons |  |  |  |  |  |  |  |  |  |
| T0 *vs*. T1 | 10 | 100.0 | 100.0 |  | 100.0 | 84.2 |  | 94.7 | 94.7 |
| T0 *vs*. T2 | 3 | 100.0 | 100.0 |  | 89.5 | 100.0 |  | 100.0 | 100.0 |
| T0 *vs*. T3 | 5 | 100.0 | 100.0 |  | 89.5 | 100.0 |  | 100.0 | 100.0 |
| T1 *vs*. T3 | 3 | 100.0 | 100.0 |  | 84.2 | 84.2 |  | 89.5 | 100..0 |
